# CYK4 Promotes Antiparallel Microtubule Bundling by Optimizing MKLP1 Neck Conformation

**DOI:** 10.1371/journal.pbio.1002121

**Published:** 2015-04-13

**Authors:** Tim Davies, Noriyuki Kodera, Gabriele S. Kaminski Schierle, Eric Rees, Miklos Erdelyi, Clemens F. Kaminski, Toshio Ando, Masanori Mishima

**Affiliations:** 1 Gurdon Institute, University of Cambridge, Tennis Court Road, Cambridge, United Kingdom; 2 Department of Physics and Bio-AFM Frontier Research Center, Kanazawa University, Kanazawa, Japan; 3 Department of Chemical Engineering and Biotechnology, University of Cambridge, Pembroke Street, Cambridge, United Kingdom; 4 Biomedical Cell Biology, Warwick Medical School, University of Warwick, Gibbet Hill Road, Coventry, United Kingdom; Vanderbilt University School of Medicine, UNITED STATES

## Abstract

Centralspindlin, a constitutive 2:2 heterotetramer of MKLP1 (a kinesin-6) and the non-motor subunit CYK4, plays important roles in cytokinesis. It is crucial for the formation of central spindle microtubule bundle structure. Its accumulation at the central antiparallel overlap zone is key for recruitment and regulation of downstream cytokinesis factors and for stable anchoring of the plasma membrane at the midbody. Both MKLP1 and CYK4 are required for efficient microtubule bundling. However, the mechanism by which CYK4 contributes to this is unclear. Here we performed structural and functional analyses of centralspindlin using high-speed atomic force microscopy, Fӧrster resonance energy transfer analysis, and in vitro reconstitution. Our data reveal that CYK4 binds to a globular mass in the atypically long MKLP1 neck domain between the catalytic core and the coiled coil and thereby reconfigures the two motor domains in the MKLP1 dimer to be suitable for antiparallel microtubule bundling. Our work provides insights into the microtubule bundling during cytokinesis and into the working mechanisms of the kinesins with non-canonical neck structures.

## Abstract 2

Cell division depends on the antiparallel bundling of microtubules by a motor complex called centralpindlin. This study reveals how the centralspindlin non-motor subunit CYK4 reconfigures the motor domains of the kinesin subunit MKLP1 to help it carry out this role.

## Author Summary

Cell division requires coordination of many different cellular components. Cytokinesis is the process by which the cytoplasm divides between the two forming daughter cells. During cytokinesis, centralspindlin is truly central, as it organizes microtubule bundle structures, recruits other factors to the site of division, and anchors the plasma membrane at the inter-cellular bridge while the two daughter cells are waiting for the final separation. Centralspindlin is a heterotetramer composed of two molecules of a kinesin-6 motor subunit, MKLP1, and two molecules of the second subunit, CYK4. For efficient microtubule bundling, both the microtubule motor subunit MKLP1 and the non-motor CYK4 subunit are required. However, it has remained unclear how CYK4 contributes to this activity. Here, we took a combinatorial approach to investigate this process, using in vitro reconstitution and structural analyses by atomic force microscopy and Förster resonance energy transfer. We revealed that the CYK4 dimer binds to a hitherto unknown globular domain at the neck of the MKLP1 dimer and optimizes the configuration of two motor domains, making them suitable for antiparallel microtubule bundling. This provides novel insight into how other kinesin superfamily molecules with non-canonical neck structures may work.

## Introduction

**Fig 1:**
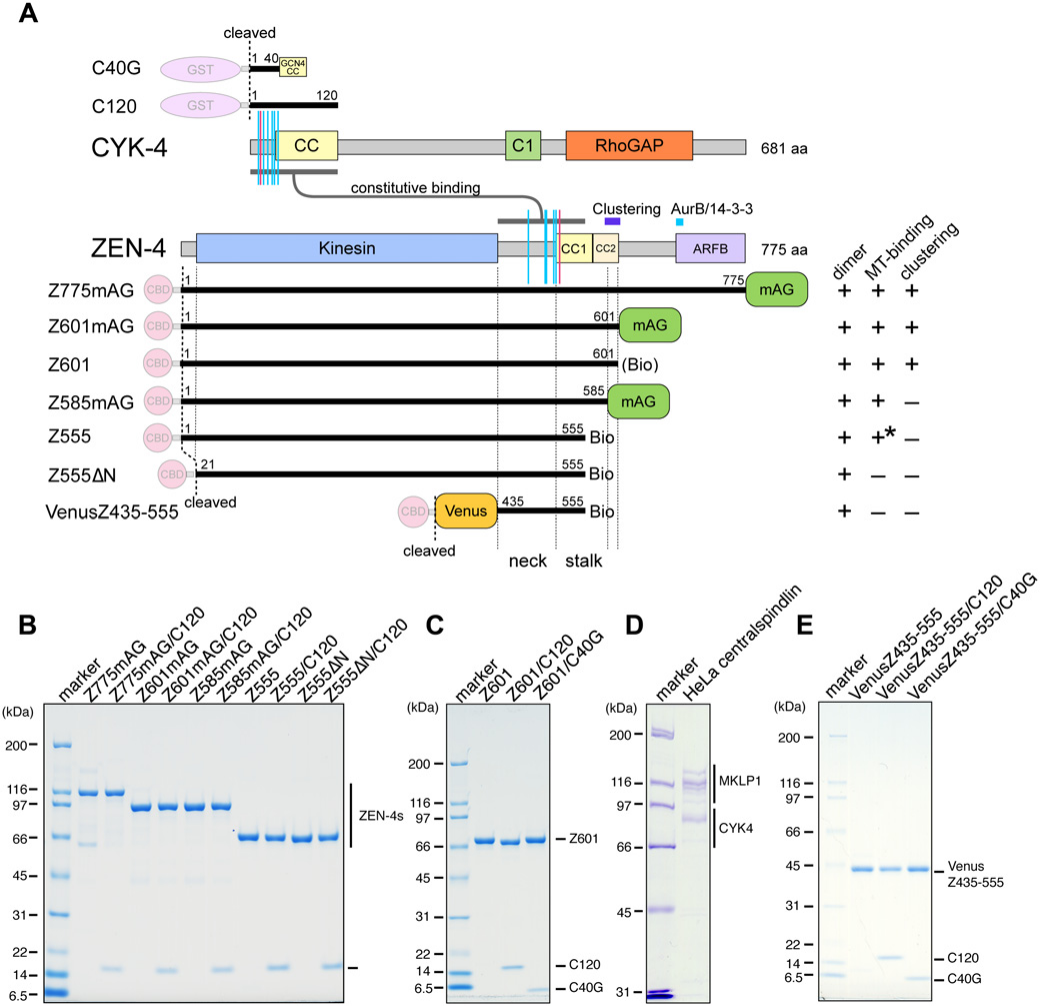
Centralspindlin preparations used in this study. (A) A schematic of the domain structures of CYK-4 and ZEN-4 and their fragments expressed as fusion proteins with purification tags, which were removed during purification. Magenta and cyan vertical segments denote the residues whose mutation disrupts the CYK-4–ZEN-4 interaction and whose mutation suppresses the interaction defect, respectively. Expected behaviors of the ZEN-4 constructs in dimerization, microtubule-binding (MT-binding) and clustering are indicated (+ or –) based on previous results [13,34], except for microtubule-binding of Z555ΔN (*), which is based on results described below. CC1, CC2: coiled coil predicted with high and low propensity, respectively; C1: C1 domain; RhoGAP: Rho-family GTPase-activating protein domain; Kinesin: kinesin motor domain; ARFB: ARF6-binding domain [9,10], BIO: 20 aa biotinylation tag. (B–E) Protein preparations used in this study were run on SDS-PAGE gels and visualized with Coomassie stain. Note that the version of Z601 used in Z601/C120, which lacks the biotinylation tag, showed a slightly larger mobility than those used alone or in a complex with C40G.

Centralspindlin is an evolutionarily conserved, constitutive 2:2 heterotetrameric complex of a kinesin-6 subunit MKLP1 and a non-motor subunit CYK4. In this article, we shall use the term “MKLP1” to refer collectively to the orthologs of the mammalian KIF23/MKLP1 [1], such as *Caenorhabditis elegans* ZEN-4, and the term “CYK4” to denote the orthologs of *C*. *elegans* CYK-4 [2], such as mammalian RACGAP1/MgcRacGAP [3]. Centralspindlin plays essential roles in cytokinesis by forming central spindle and midbody microtubule bundle structures, by recruiting and regulating various factors at the site of division, and by anchoring the plasma membrane in the intercellular bridge while the daughter cells are waiting for abscission [4–11]. A point mutation in KIF23/MKLP1 is the cause of congenital dyserythropoietic anemia type III, which is characterized by large multinucleated erythroblasts in bone marrow [12]. Both the MKLP1 and CYK4 subunits are essential for microtubule bundling by centralspindlin [3,13]. In vitro, neither MKLP1 alone nor CYK4 alone can efficiently bundle microtubules. In vivo, depletion of either component or point mutations that affect the formation of the centralspindlin heterotetramer cause the central spindle defects [2,3,14–19]. Strikingly, although genetic screens in *C*. *elegans* for suppressors of such complex-disrupting mutations (S15L in CYK-4 or D520N in ZEN-4) so far identified 15 independent point mutations, all reside within limited regions; CYK-4 12–39 and ZEN-4 477–515. It is likely that these findings define the binding interfaces between these subunits [3,13] (summarized in Fig 1A). These are included in the minimal domains of CYK-4 and ZEN-4 sufficient for in vitro reconstitution of the stable complex between them (CYK-4 1–120 and ZEN-4 435–555). These data emphasize the importance of heterotetramer formation for microtubule bundling and suggest that the tetramer assembly is achieved through compact domains without extensive contact, such as a long four-helix bundle. However, it remains unclear how CYK4 contributes to microtubule bundling.

Although MKLP1, a kinesin-6 [20], has the kinesin motor domain at its N-terminus, it is distinct from other N-terminal kinesins (N-kinesins). In kinesins-1, -2, -3, -4, -5, and -7, the catalytic core, which contains an ATP-binding pocket and a microtubule-binding surface, is connected to the coiled coil via a short conserved sequence motif of about 15 aa, so-called the "neck linker" [21–23]. The neck linker docks to the catalytic core in a manner bidirectionally coupled to the nucleotide and microtubule-binding states of the catalytic core. Thus, it plays crucial roles in force-generation by individual motor domains and in the mechanochemical coordination between the two heads in walking kinesin dimers. Interestingly, MKLP1 does not have a recognizable neck linker. Instead, the catalytic core of MKLP1 is connected to the coiled coil via a longer "neck" sequence of 60–70 aa, which contains dispersed helix/coil-breaking proline residues (“MKLP1” in S1 Fig) [3]. Importantly, this neck region of MKLP1 contains the CYK4 binding site. Because of these unusual mechanistic features, the molecular structure of the heterotetrameric centralspindlin complex of CYK4 and MKLP1 is of great interest. Although a recent study using electron paramagnetic resonance spectroscopy reported a change in the mobility of ZEN-4 neck residues upon CYK-4 binding [24], it remains unclear how this influences the configuration of the two motor domains and how this modulates microtubule bundling by the complex.

Here we performed direct visualization of the dynamic structure of centralspindlin by atomic force microscopy. Our data indicate that CYK4 binding to the neck domain of the MKLP1 dimer remodels the configuration between the two motor domains. Furthermore, by using in vitro functional assays, we demonstrate that this reconfiguration optimizes the centralspindlin complex for the formation of and accumulation to the antiparallel microtubule bundles crucial for cytokinesis.

## Results

### High-Speed AFM Identifies a Globular Mass at the Neck Region of ZEN-4/MKLP1

Atomic force microscopy (AFM) is unique in its capability to capture high-resolution images of intact biological samples in aqueous solution without chemical fixation or staining [25–27]. Recent dramatic improvement of the temporal resolution achieved by high-speed AFM (HS-AFM) enables direct observation of dynamic protein behaviors such as conformational change and association/dissociation [28–30]. Here, we utilized this technique to visualize how CYK4 is complexed with MKLP1.

**Fig 2:**
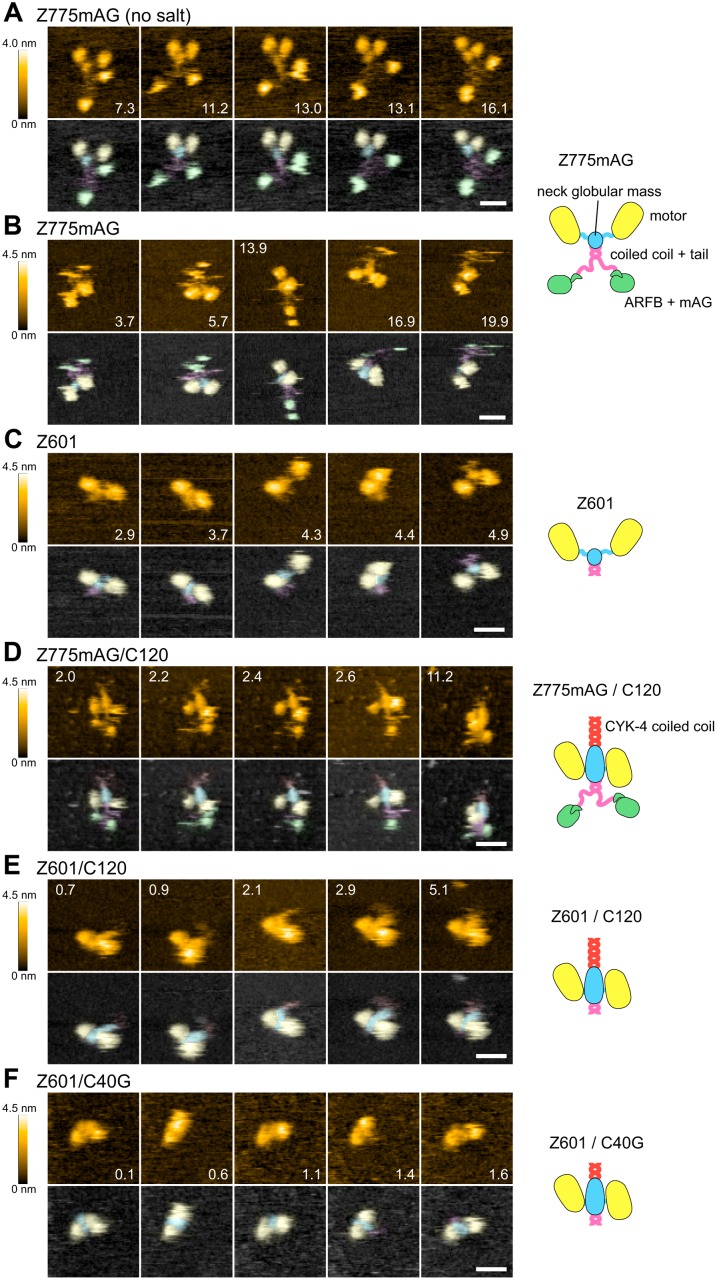
CYK-4 binds to the neck globular mass of ZEN-4. (A–F) Centralspindlin constructs were observed by high-speed atomic force microscopy (HS-AFM). Areas of 70×70 (A, B) or 60×60 (C–F) nm^2^ squares were imaged in 80×80 pixels every 149 (A), 100 (B, C, E, F), or 200 (D) ms. Images of identical molecules at the indicated time points (s) taken from the corresponding movies are displayed (A: S1 Movie, B: S2 Movie, C: S3 Movie, D: S4 Movie, E: S5 Movie, and F: S6 Movie; see S1 Text for detailed specifications). Top row in each panel: height from the mica surface color-coded in orange (scales on the left). Bottom row in each panel: domain assignment based on comparison of these constructs is shown in pseudocolor as in the associated schematics (yellow: ZEN-4 motor domain, cyan: neck globular mass, magenta: ZEN-4 coiled coil and flexible tail, green: ARFB+mAG, red: CYK-4 coiled coil). Scale bars, 20 nm.

We started by observing full-length *C*. *elegans* ZEN-4 kinesin tagged with a green fluorescent protein, monomeric Azami Green (Z775mAG, Fig 1A and 1B) [31,32], adsorbed onto a mica surface. Under a salt free condition (Fig 2A, S1 Movie), which would promote adsorption of the protein to the mica surface and thus allow a better resolution, we observed that the tagged ZEN-4 molecule consists of a pair of globular domains (pseudocolored in yellow in the bottom rows), a nearby smaller globular mass (cyan), a short rod-like structure (magenta), flexible linkers (magenta), and another pair of fluctuating globular domains (green). Other representative examples of independent molecules of Z775mAG under this condition are found in S3 Fig and S7 Movie. In the presence of a slightly increased salt (Fig 2B and S2 Movie; S3 Fig and S7 Movie for other representative images), which would minimize artificial extension of the flexible protein molecules that might be caused by excess adsorption to the surface, the images of the molecules became somewhat less clear due to the increased mobility. Nonetheless, we could detect the same features, although the fluctuating globular domains (pseudocolored green) now appeared smaller than the pair of the globular domains (yellow) with a nearby small globular domain (cyan). Hereafter, we used this condition to better preserve dynamic inter-domain configurations.

Z775mAG has a globular kinesin domain at its N-terminus. A small globular domain at the ZEN-4 C-terminus [9] tagged with a fluorescent protein moiety would also appear as a single globular mass by AFM. To assign these to the molecular features observed above, we then observed a ZEN-4 construct, Z601, which lacks both the C-terminal tail and the mAG tag (Fig 1A and 1C). Similarly to Z775mAG, we could detect the two large globular domains (yellow) with a nearby smaller globular mass (cyan) in Z601 (Fig 2C, S3 Movie; S3 Fig and S7 Movie for other representative images). On the other hand, the highly mobile globular domains (green) that were linked to the other part of the Z775mAG molecule via a flexible linker (magenta) were not detected. Thus, we conclude that these missing parts, i.e., the majority of the flexible linker and the highly mobile globular domains, correspond to the C-terminal tail of ZEN-4 and the mAG tag. This is consistent with the prediction that about two-thirds of the ZEN-4 tail (around aa 600 to 710) would be unfolded [33].

The parts commonly detected in Z775mAG and Z601 should correspond to ZEN-4 1–601, which include the kinesin motor domain (aa 20–434), regions predicted to be parallel coiled coils with high (aa ~530 to ~565, CC1) or low (aa ~566 to ~600, CC2) probabilities and the neck domain that links these parts (aa 435 to ~530) (Fig 1A). A motor domain (415 aa) should form a globular domain, which is bigger than the other parts in a Z601 dimer combined (166 aa × 2). Thus, the two larger globular domains (yellow) should be assigned to the ZEN-4 motor domains. The smaller mobility of these domains than the C-terminal tail domain plus the mAG tag, especially in the absence of salt, might be due to stronger adsorption of the positively charged microtubule-binding surface to the negatively charged mica surface under this condition. Interestingly, in both Z775mAG and Z601, we observed an unexpected globular mass (cyan) at the interface between the two motor domains and the flexible linker (magenta), which largely disappeared in Z601 except for a short remnant. This indicates that at least a part of the long ZEN-4 neck sequence (aa 435 to ~530) folds into a globular structure (cyan) as, otherwise, the two motor domains would appear to be linked by long and flexible linkers similar to those in the C-terminal tail (around aa 600 to 710).

### CYK4 Binds to the Neck Globular Mass in MKLP1

We then proceeded to analyze the complex of CYK-4 and ZEN-4. CYK-4 1–120 (C120), the minimum fragment of CYK-4 that is able to dimerize and form a complex with ZEN-4, was coexpressed with Z775mAG in bacteria, co-purified through affinity purification steps and gel filtration (Fig 1B and S2 Fig for co-elution from a gel filtration column) and subjected to HS-AFM (Fig 2D and S4 Movie; S3 Fig and S7 Movie for other examples). In Z775mAG/C120, all the parts found in Z775mAG alone, such as the motor domains (yellow), the neck globular mass (cyan), and the ZEN-4 C-terminal tail plus mAG, were commonly detected. In addition, a flexible tail-like structure (red) was occasionally observed protruding from the neck globular mass (cyan) on the opposite side of the flexible tail linker (magenta) in Z775mAG/C120. As this was never observed in Z775mAG alone, we ascribe this structure to the coiled coil (predicted to be aa ~40 to ~120) of CYK-4. The neck globular mass in the complex appeared bigger than that found in Z775mAG without C120, suggesting that the N-terminal non-coiled coil piece of CYK-4 (aa 1 to ~40) also contributes to this globular mass at the center of the molecule. Consistent with this assignment, a similar protrusion and increased size of the neck globular mass were observed also in a complex of Z601 with C120 (Z601/C120, Fig 2E and S5 Movie. S3 Fig and S7 Movie for other examples). Furthermore, this protrusion was hardly detectable in a complex with a chimera between the N-terminal 40 amino acids of CYK-4 and a shorter (~20 aa) coiled coil from a leucine-zipper transcription factor, GCN4 (C40G) (Fig 2F and S6 Movie; S3 Fig and S7 Movie for other examples) [13] although co-elution in size exclusion chromatography confirmed the stable complex formation between Z601 and C40G (S2B Fig). This verifies our assignment that the protrusion that appears only when ZEN-4 is in a complex with C120 corresponds to the coiled coil of CYK-4. The lower visibility of the predicted ZEN-4 coiled coil in Z601 constructs than that of CYK-4 might indicate that it would be actually shorter (the region predicted with high probability is only as short as ~35 aa, Fig 1A, CC1) or partly embedded within the neck globular domain.

In the Z775mAG/C120 complex, the CYK-4 coiled coil showed direct contact neither with the ZEN-4 motor domains nor the ZEN-4 coiled coil (Fig 2D and 2E; see also S4 Movie, S5 Movie, and S7 Movie). This is consistent with the genetic and biochemical data [3,13] that define the interaction interfaces as the N-terminal piece of CYK-4 and the neck domain of ZEN-4. The linear arrangement of CYK-4 coiled coil, the globular mass made of the CYK-4 N-terminal piece and the ZEN-4 neck domain, and the ZEN-4 coiled coil form a molecular backbone in a dagger-like appearance. ZEN-4 motor domains tended to be positioned on the opposite sides of this molecular backbone, although occasionally they were observed on the same side (AFM image at 11.2 s in Fig 2D, S3 Fig, S7 Movie).

**Fig 3:**
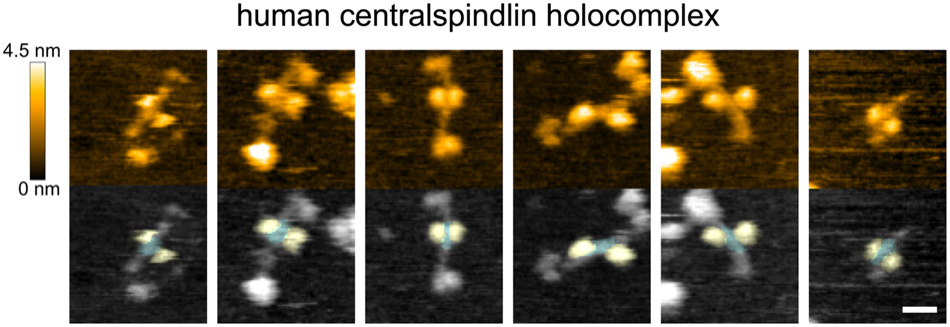
Molecular architecture of centralspindlin is evolutionarily conserved. Centralspindlin holocomplex purified from HeLa cells was observed by HS-AFM. 80×80 nm^2^ squares were imaged in 80×80 pixels every 215 ms. Although the overall shape of the molecules in this preparation was more heterogeneous than the *C*. *elegans* centralspindlin constructs expressed in bacteria, a molecular architecture similar to that of *C*. *elegans* proteins was commonly observed, i.e., a pair of globular domains (yellow) on opposite sides of a linear molecular axis with a central globular domain mass (cyan). Scale bar, 20 nm.

Finally, to examine whether the molecular architecture of centralspindlin described above is conserved across species, we observed the human centralspindlin holocomplex purified from HeLa cells (Fig 1D, Fig 3, S8 Movie and S9 Movie) [34]. Human centralspindlin also had a long molecular backbone with a central globular mass, at which the two motor domains reside on the opposite sides. In addition, globular masses were observed at the both ends of the molecular backbone although the shapes and sizes are variable. This probably reflects the heterogeneity of this preparation due to the alternative splicing within the C-terminal tail of MKLP1 [35], post-translational modifications, interacting proteins, and partial degradations during the purification steps. In human holocomplex, rod-like structures were detected on both sides of the central globular mass (cyan) along the molecular axis (Fig 3). This is consistent with the fact that human MKLP1/KIF23 has a region of high propensity for a coiled coil (aa 535 to 660) about 2-fold longer than ZEN-4 [33].

In summary, HS-AFM revealed the unique, evolutionary conserved, molecular architecture of centralspindlin. A part of the neck domain of the MKLP1 subunit of centralspindlin folds into a globular domain, which provides the binding site for the N-terminal non-coiled coil region of the CYK4 subunit. Via this interface, the homodimers of CYK4 and MKLP1, both of which were dimerized as parallel coiled coils, assemble into a heterotetramer, forming a linear molecular backbone.

### CYK4 Subunit Confers Antiparallel Preference to the Microtubule Bundling by Centralspindlin

To understand the implications of this molecular architecture of centralspindlin on its function, we then performed in vitro reconstitution experiments. Due to specific experimental design requirements for each assay as detailed below, we chose suitable dimeric ZEN-4 constructs with various C-terminal deletions. However, the common mode of CYK-4 binding to both Z775mAG and Z601 observed above independently of the C-terminal tail suggests that CYK-4 binding should influence the structure of ZEN-4 in an identical manner across the different assays.

**Fig 4:**
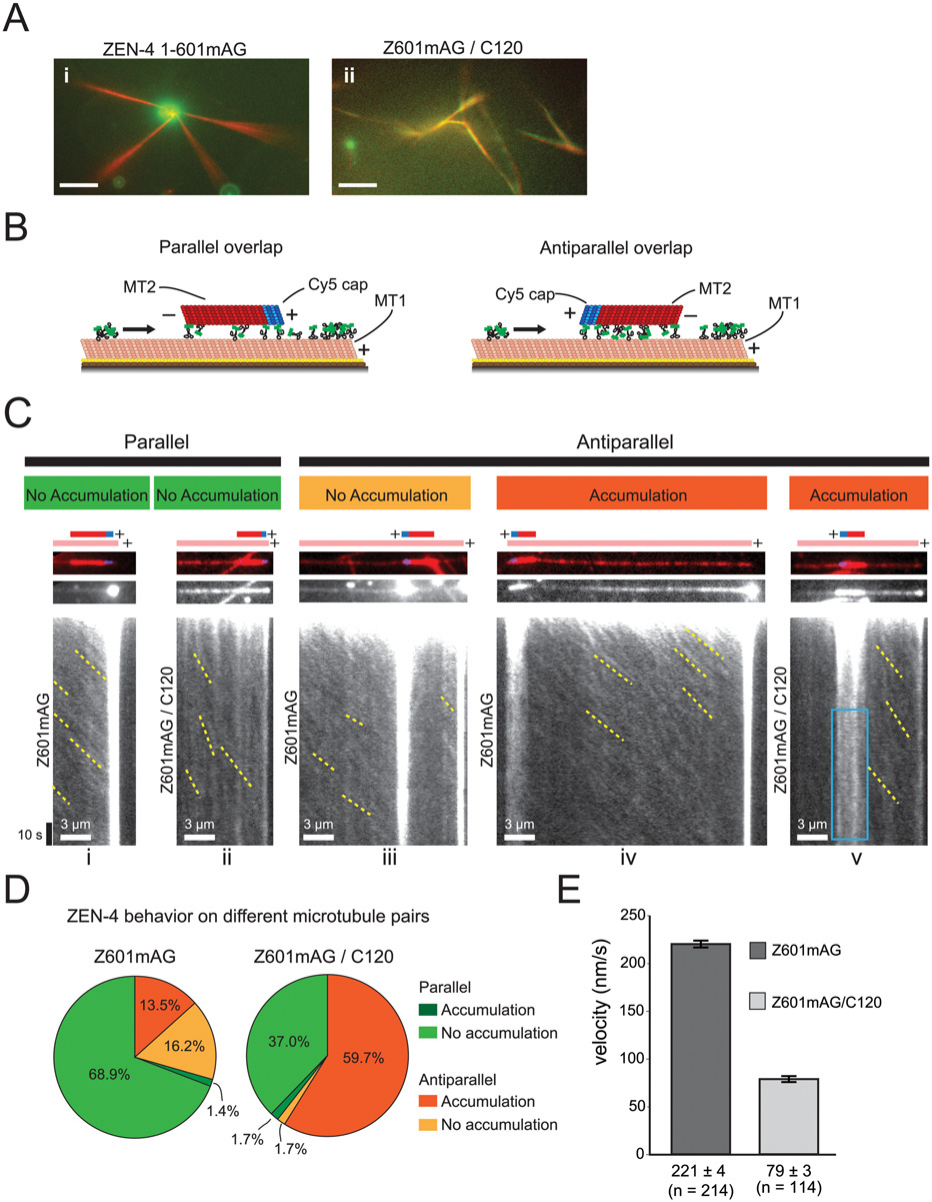
CYK-4 is required for antiparallel microtubule bundling by centralspindlin. (A) Merged fluorescence microscope images of rhodamine microtubules (red) and ZEN-4 tagged with mAG (green). (i) Z601mAG organized the microtubules into aster-like structures with intense mAG signal at the center. (ii) Tetrameric Z601mAG/ C120 coated the microtubules and caused small microtubule bundles to form. 200 nM of ZEN-4 1–601mAG; 25 mM KCl; scale bar, 5 μm; images collected 10–30 min after mixing. (B) Scheme of the experimental setup. Biotinylated and dimly rhodamine-labelled microtubules (MT1) were immobilized on the coverslip via Neutravidin–biotin interactions. Z601mAG and Z601mAG /C120 were perfused into the flow cell with non-biotinylated and brightly rhodamine-labeled microtubules with a Cy5 polarity mark at their plus-ends (MT2). The free MT2 could land on the immobilized MT1 and, in some cases, accumulate the ZEN-4 complexes. By observing the direction of ZEN-4 movement and plus end accumulation, the polarity of the immobilized microtubule could be determined. (C) Kymographs showing representative examples of the five commonly observed overlap types. The schematics at the top of each panel indicate the position and orientation of the overlaps and are based on imaging the rhodamine (red) and Cy5 (blue) channels. The central panels show the mAG channel at the first time point. The lower panels are kymographs showing movement and accumulation of the mAG labeled motor complexes. Both Z601mAG and Z601mAG/C120 move to the plus end. Yellow dotted lines indicate particle movement. The blue box encloses a region where particles can be seen to move bidirectionally on the antiparallel overlap. (D) Pie charts showing Z601mAG and Z601mAG/C120 accumulation frequencies. When dimeric Z601mAG was observed, the most common scenario was for parallel overlaps on which ZEN-4 1–601mAG did not accumulate (69%, *n* = 74). If accumulation did occur, it was independent of the polarity of microtubule orientation. In contrast, Z601mAG/C120 showed a bifurcate accumulation distribution, either accumulating on antiparallel microtubules (63%) or not accumulating on parallel microtubules (34%, *n* = 119). (E) Motility was measured using kymographs along immobilized microtubules. The Z601mAG sample showed a velocity of 221 ± 4 nm/sec while this was reduced in Z601mAG/C120 to 79 ± 3 nm/sec (mean ± standard error [SE])

Although it has been shown that the C120 can reconstitute the heterotetramer, as we confirmed at high resolution by AFM, it has not been directly tested whether this CYK-4 fragment is sufficient to reconstitute microtubule-bundling activities in a complex with ZEN-4, a critical role of centralspindlin in vivo. Previous studies demonstrated that centralspindlin oligomerizes into higher-order assemblies depending on a short motif (aa 586 to 601 in ZEN-4) within a region predicted to be a coiled coil with low propensity (CC2, Fig 1A) and that this clustering is required for continuous single particle motility and efficient microtubule bundling [34]. Without CYK-4, the minimal ZEN-4 motor construct that is able to form clusters, ZEN-4 1–601 tagged with mAG (Z601mAG, Fig 1A), was able to crosslink taxol-stabilized microtubules in vitro and organize them into aster-like structures (Fig 4A i). Z601mAG strongly accumulated in the center of the aster-like structures. In contrast, the same ZEN-4 construct in a complex with C120 (Z601mAG/C120) formed microtubule bundles similar to those made by centralspindlin complexes containing longer forms of CYK-4 (Fig 4A ii) [3,13,34]. On these bundles, Z601mAG was distributed throughout the microtubule overlaps, as expected for a microtubule-bundling factor (Fig 4A ii). These observations show that C120 in a complex with ZEN-4 is sufficient to reconstitute the microtubule-bundling activity of the full-length centralspindlin complex.

In dividing cells, centralspindlin strongly accumulates in the spindle midzone, where two sets of interpolar microtubules originating from the opposite spindle poles overlap with their plus-ends, interdigitating in an antiparallel manner. To examine the polarity of the microtubule bundles formed by centralspindlin, we set up an in vitro assay in which the polarity of two single microtubules in a bundle can be determined (Fig 4B). In this system, first, biotinylated and dimly rhodamine-labeled microtubules (MT1) are immobilized on a coverslip via avidin. Following this, non-biotinylated and brightly rhodamine-labeled microtubules with a plus-end mark by Cy5 (MT2) are introduced to the chamber, in the presence of either dimeric Z601mAG, or heterotetrameric Z601mAG/C120, both of which can oligomerize into clusters [34]. Polarity of MT1 was determined by the plus-end-directed movement of Z601mAG or Z601mAG/C120 particles along them and by the accumulation of these motors at their plus-ends (Fig 4C). Interestingly, ZEN-4 in complex with CYK-4 (Z601mAG/C120) preferentially formed antiparallel overlaps (Fig 4C v and Fig 4D) while ZEN-4 alone (Z601mAG) typically formed parallel bundles (Fig 4C i and Fig 4D). Both types of the bundle were static, with no observable microtubule sliding for up to 10 min of observation although we cannot exclude the possibility that the sliding might occur at the very early phase of bundling, before observation began. Importantly, we observed that Z601mAG/C120 specifically accumulates on antiparallel microtubule pairs (Fig 4C v and Fig 4D). On parallel microtubule pairs, Z601mAG/C120 continued to move to the plus end and did not accumulate on the zone of overlap (Fig 4C ii). Accumulation of Z601mAG/C120 on the antiparallel overlap is not just due to the polarity of the overlap but rather requires C120 for this specificity. This is clear, as less than half of the antiparallel overlaps made by Z601mAG alone showed such accumulation (Fig 4C iii versus iv, Fig 4D). Observing the behavior of Z601mAG/C120 at the overlaps on the level of a single molecule is difficult due to high density of fluorescent proteins. However, because many of these bleached during observation, at later time points it was possible to detect both static particles and those showing bidirectional movement (Fig 4C v; blue box), indicating that the bundles made by centralspindlin are not totally static. Furthermore, we measured the velocity of particle movement along the microtubules. We found a large difference between Z601mAG and Z601mAG /C120, with velocities of 221 ± 4 nm/sec and 79 ± 3 nm/sec respectively (mean ± SE, *n* = 214 and 114) (Fig 4E). Together, these results indicate that the N-terminal piece of CYK-4 is sufficient to reconstitute a complex with ZEN-4 that has the ability to bundle microtubules in an antiparallel manner and to accumulate on the antiparallel overlaps, as observed in vivo. In addition, the CYK-4 modifies ZEN-4 motility on microtubules.

### CYK4 Modifies the Motor Properties of MKLP1

**Fig 5:**
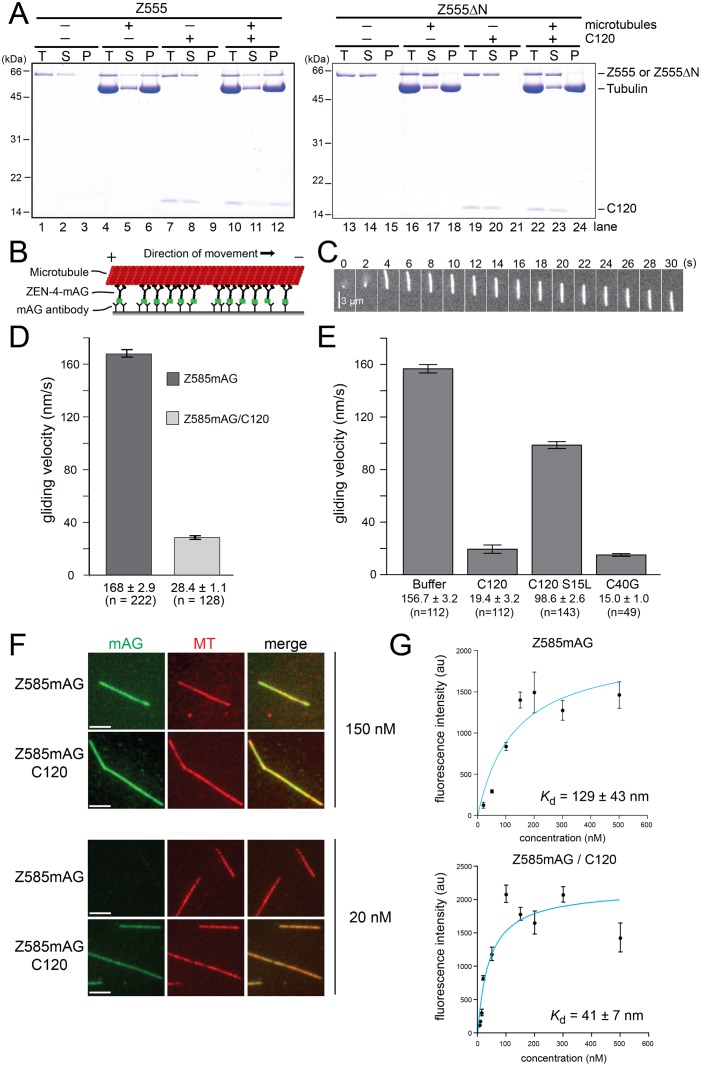
CYK-4 modulates the interaction between ZEN-4 motor and microtubules. (A) ZEN-4 co-sedimentation with taxol-stabilized microtubules. In vitro microtubule pull-down assays were performed with recombinant Z555 and Z555ΔN as homodimers and also in the tetrameric form with C120. The total (T), supernatant (S), and pellet (P) fractions were collected, analyzed by SDS-PAGE, and stained with Coomassie blue. Z555 and Z555/C120 sedimentation only occurs when bound to microtubules. In contrast, Z555ΔN failed to do so irrespective of the presence of C120, indicating that C120 itself does not bind directly to microtubules. (B) Scheme showing the surface gliding assay. The C-terminal mAG epitope on Z585mAG is immobilized on the coverslip via anti-mAG antibody. Plus-end directed motility of the motors results in gliding of microtubules with their minus-end in front. (C) Fluorescence microscopy showing a Rhodamine-labeled microtubule land on the coverslip surface and then move across the field of view. (D) Microtubule gliding velocity of Z585mAG purified as dimeric Z585mAG or tetrameric Z585mAG/C120. The tetrameric complex has dramatically reduced gliding velocity. (E) Dimeric Z585mAG was incubated with either C120 or the artificially dimerized CYK-4 1–40 construct (C40G) for 10 min prior to immobilization. Microtubule-gliding velocity was significantly reduced by both C120 and C40G. Gliding velocities are displayed as the mean ± SE. *P*-value determined using Wilcoxon rank-sum test. (F) Z585mAG dimer and 585mAG/C120 were bound to immobilized rhodamine microtubules at a range of concentrations in 1 mM AMP-PNP. At higher concentrations (150 nM), the dimeric and tetrameric complexes showed similar intensity on the microtubules. At low concentrations (20 nM), Z585mAG bound more weakly than Z585mAG/C120. Identical laser power was used for all imaging. Display settings are conserved for panels showing the same motor concentration. (G) The mean intensity at the range of motor concentrations was plotted and used to determine the dissociation constant (*K*_d_) (see Materials and Methods), showing Z585mAG/C120 has a higher affinity for microtubules (*K*_d_ = 41 nM) than Z585mAG (*K*_d_ = 129 nM). 25 mM NaCl; 1 mM AMP-PNP; scale bar, 3 μm.

How does CYK4 contribute to the antiparallel microtubule bundling? A simple possibility is that CYK4 provides an additional site for microtubule binding, allowing bridging between two microtubules by the centralspindlin complex. Alternatively, CYK4 may not directly interact with microtubules itself, but instead regulate how MKLP1 interacts with microtubules. To discriminate between these possibilities, we tested whether C120 has an ability to directly bind to microtubules independently of ZEN-4 using a microtubule sedimentation assay (Fig 5A). Crucial for this type of assay is the solubility of the samples to be examined for binding to microtubules under the buffer and centrifugation conditions that should precipitate the majority of microtubules irrespective of their assembly state (e.g., dispersed, cross-linked, or bundled). C120 alone could not be used because, without ZEN-4, it forms insoluble aggregates. Instead, we evaluated the microtubule interaction of C120 by comparing the microtubule-binding activity of the complexes between C120 and soluble ZEN-4 variants with full or severely reduced microtubule-binding activity. To achieve maximal solubility and to minimize cross-linking and/or bundling of microtubules, which would obscure the interpretation, we used ZEN-4 constructs lacking the clustering domain [34,36]. ZEN-4 has a highly positively charged tail at its N-terminus, which is required for efficient interaction with microtubules [37]. As expected, co-sedimentation with microtubules in the presence of the nonhydrolyzable ATP-analog, AMP-PNP, was largely abolished by removing this N-terminal tail (ZEN-4 1–555 [Z555] versus ZEN-4 21–555 [Z555ΔN], Fig 5A lanes 5 and 6 versus lanes 17 and 18). The complex of C120 with Z555 co-purified from bacteria (S2C Fig) was soluble (lanes 8 and 9) but co-sedimented with microtubules (lanes 11 and 12) in a similar manner to Z555 alone. In contrast, the complex lacking the ZEN-4 N-terminal tail (C120/Z555ΔN) did not co-sediment with microtubules, but remained in the supernatant (lanes 23 and 24). This indicates that the microtubule-binding activity of the CYK-4 and ZEN-4 complex largely depends on the interaction of ZEN-4 with microtubules and the interaction between CYK-4 and microtubules is not significant.

The above result suggests that it is unlikely that CYK-4 contributes to the microtubule bundling by binding microtubules. On the other hand, the velocity of the motility of Z601mAG particles was slower in the presence of C120 (Fig 4E). Thus, it is plausible that CYK-4 plays a role in modifying the interaction between ZEN-4 and microtubules. To investigate this, we examined whether CYK-4 influences the intrinsic properties of ZEN-4 as a molecular motor in two different ways.

First, we measured the motility of different ZEN-4 constructs using surface gliding assays, which, in contrast to the bundling assays, do not require clustering of ZEN-4. We immobilized a soluble and dimeric construct of ZEN-4 (Z585mAG, Fig 1A and 1B) on a glass surface using a C-terminal mAG tag (Fig 5B). This exhibited microtubule movement at a velocity of 180 nm/s (Fig 5C). Strikingly, when we used ZEN-4 co-purified with C120, we observed an even more dramatic reduction of the gliding velocity, to 30 nm/s (Fig 5D), than we saw in the motility of Z601mAG particles along immobilized microtubules (Fig 4E). This could be reproduced by incubation of ZEN-4 with C120 prior to the assay, under the condition in which C120 is kept soluble (Fig 5E, “C120”), showing that the decreased motility of the Z585mAG/C120 complex was not due to inactivation during the purification procedure. A point mutation (S15L) on the N-terminal piece of CYK-4, which greatly affects the affinity to ZEN-4 [3,13], largely abolished the effect of CYK-4 on the ZEN-4 motility (Fig 5E, “C120 S15L”), indicating the specific requirement of the interaction between CYK-4 and ZEN-4. In contrast, artificially dimerized N-terminal 40 aa of CYK-4 (C40G, Fig 1A) was able to decrease the motility of ZEN-4 (Fig 5E, “C40G”). These data indicate that the N-terminal 40 aa of CYK-4, if it is dimerized, is sufficient for modifying the motor activity of ZEN-4 and that the specific amino acid sequence in the CYK-4 coiled coil is not important.

Second, we measured the effect of CYK-4 on the microtubule-binding affinity of ZEN-4. Fluorescently labeled ZEN-4 (ZEN-4 1-585mAG, Z585mAG) alone or as a complex with C120 (Z585mAG/C120) was introduced into a chamber containing immobilized microtubules in the presence of AMP-PNP. This resulted in uniform binding all along the microtubule, and the fluorescence intensity was measured (Fig 5F). By titrating in different ZEN-4 concentrations, the binding affinity between ZEN-4 and microtubules was determined (Fig 5G). The microtubule-binding affinity of Z585mAG/C120 (*K*_d_ = 41 ± 7 nM) was three times higher than that of dimeric Z585mAG alone (*K*_d_ = 129 ± 43 nM). These data indicate that CYK-4-binding actively modifies the kinesin activity of ZEN-4 in the centralspindlin complex in a way suitable for microtubule bundling.

### CYK4 Controls the Inter-Domain Configuration of the MKLP1 Motor Domains within Centralspindlin

**Fig 6:**
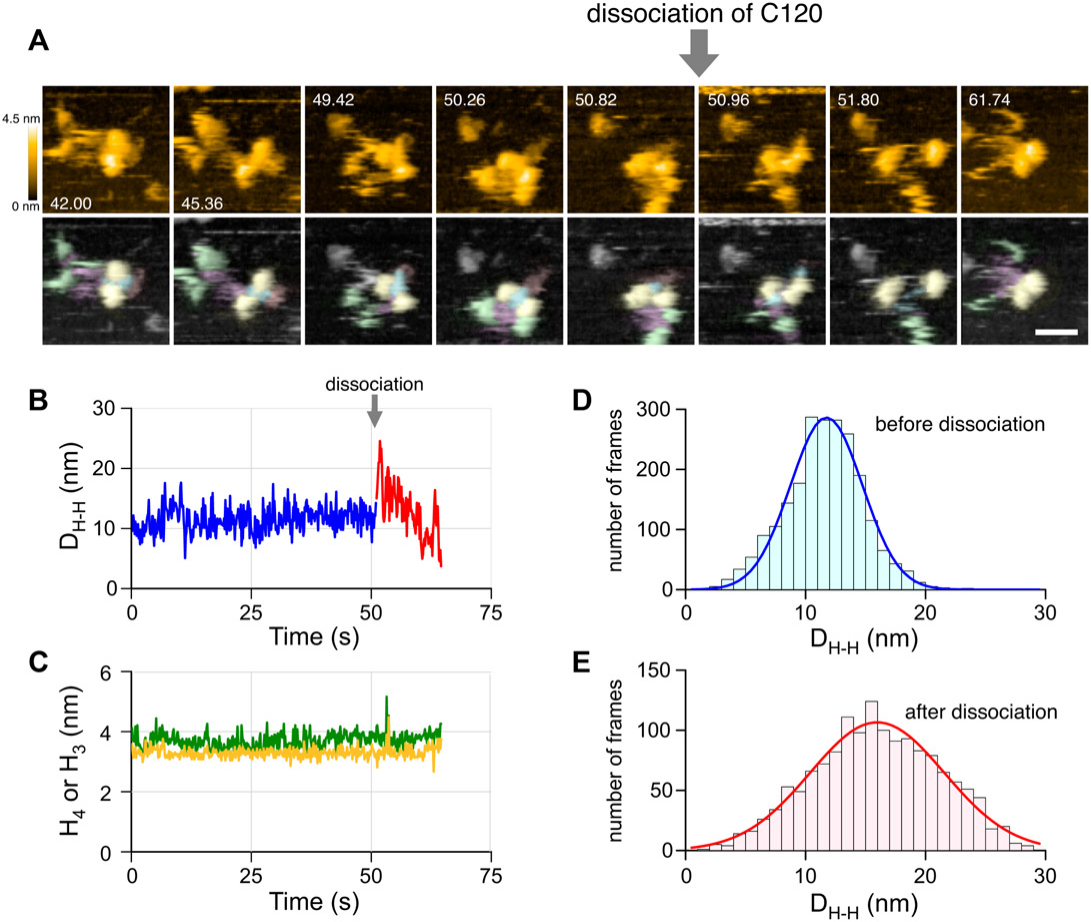
CYK-4 confines the configuration of the two ZEN-4 motor domains to be closer and less flexible. (A) HS-AFM images showing a dissociation event of C120 from Z775mAG, pseudocolored in the same way as in Fig 2 (stills taken from S8 Movie). The dissociation of C120 occurred at 50.96 s. Frame rate, 140 ms/frame; scanning area, 60×60 nm^2^ with 80×80 pixel; Z-scale, 4.5 nm; scale bar, 20 nm. (B) Time course of the head-to-head distance of ZEN-4 motor domains (D_H-H_). Blue and red lines represent the time course of D_H-H_ before and after C120 dissociation, respectively. (C) Time courses of the heights of ZEN-4 motor domains. The higher and lower height values are separately plotted into the time course as green and orange lines, respectively. Data in (B) and (C) were obtained from the molecule seen in (A). (D) and (E) Distributions of D_H-H_ before and after C120 dissociation, respectively. Solid lines represent single-Gaussian fit results. The summary of head-to-head distance measurement of this and other constructs is shown in S4E Fig.

To determine how CYK4 modulates the mode by which MKLP1 interacts with microtubules, we examined the effect of CYK-4 on the configuration of the motor domains in the heterotetrameric complex. In high-speed AFM, the detachment of CYK-4 from ZEN-4 was sometimes observed (Fig 6A and 6B, S10 Movie). This provides us with an ideal opportunity to examine the difference between the inter-head configurations within a ZEN-4 dimer in the presence and absence of CYK-4. Before dissociation of C120 from Z775mAG, the head-to-head distance (i.e., the distance between the centers of mass of ZEN-4 motor domains [D_H-H_]) was 12 ± 3 nm (mean ± SD), whereas, after dissociation, the mean D_H-H_ increased to 16 nm, with a larger standard deviation of 6 nm (Fig 6D and 6E; for additional movies and head-to-head distance measurements, S11 Movie and S4A–S4C Fig, respectively). In contrast, the heights of the ZEN-4 heads were constant (Fig 6C). Similar effect of CYK-4 on the ZEN-4 head-to-head distance was observed in Z601 constructs. Both the mean distance and its standard deviation in the complexes with C120 and C40G (10 ± 2 and 11 ± 2 nm, respectively) were smaller than that in Z601 without CYK-4 (13 ± 4 nm). Other geometrical parameters were summarized in S4E Fig. These observations indicate that CYK-4 introduces a restriction in the configuration of the two motor domains of ZEN-4 through binding onto its neck domain.

**Fig 7:**
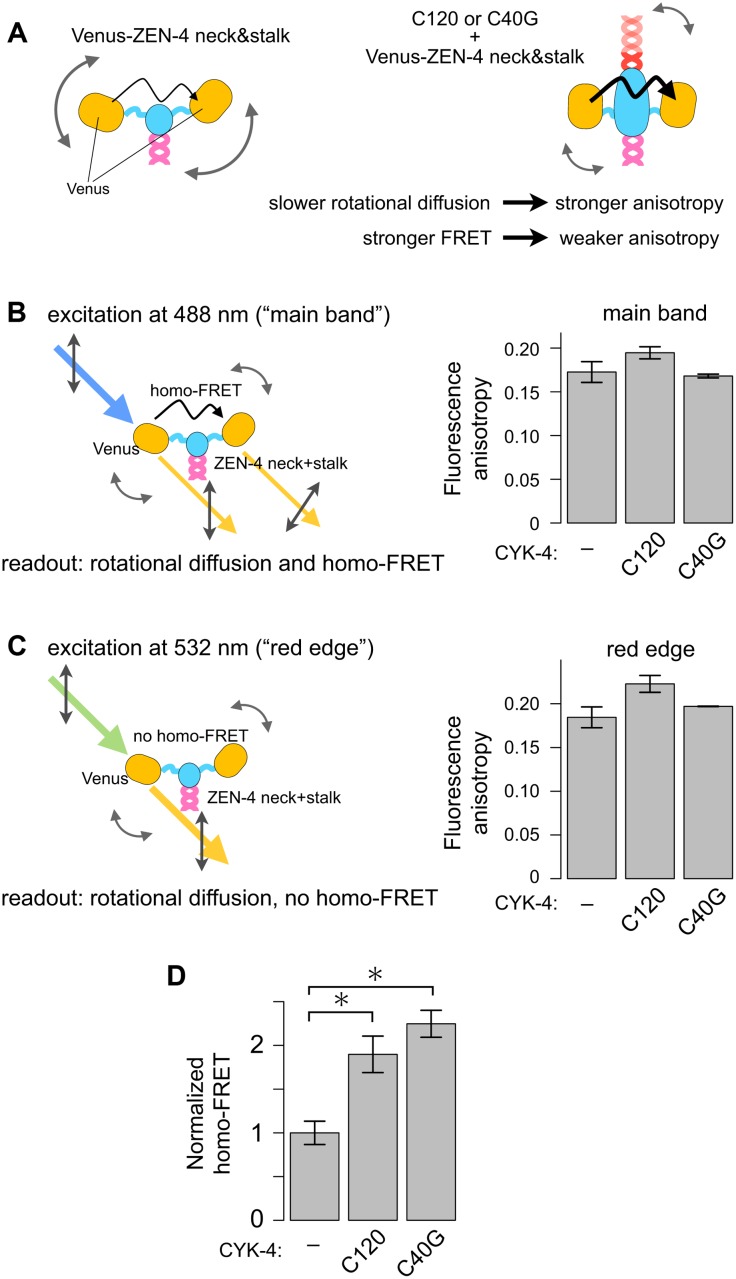
CYK-4 induces conformational change of the neck domain of ZEN-4 dimer. (A) Possible effects of CYK-4 on the fluorescence polarization anisotropy of the two Venus moieties, with which the motor domains of a ZEN-4 dimer were replaced. Slower rotational diffusion would increase the anisotropy, while the stronger Fӧrster resonance energy transfer between the two Venus moieties (homo-FRET) would decrease it. (B, C) Fluorescence anisotropy of VenusZ435-555 without CYK-4 or in a complex with C120 or C40G was measured at 488 nm (B) or 532 nm (C). (B) Fluorescence anisotropy with excitation at 488 nm (“main band excitation”) is influenced both by rotational diffusion and homo-FRET. (C) Lack of homo-FRET by excitation at 532 nm (“red edge excitation”) allows us to estimate the effect of CYK-4 binding on the rotational diffusion independently of that on the homo-FRET. (D) FRET efficiency, *E*, of the homo-FRET between the Venus-moieties was calculated by *E* = 1—*r/r0*, where *r* and *r0* are the anisotropies by main-band (B) and red-edge (C) excitations, respectively [41]. Values normalized with that of VenusZ435-555 without CYK-4 are shown. * indicates *p* < 0.01 (ANOVA corrected for multiple comparison, *n* = 6, 7, and 5). Error bars, standard error.

The above observation by AFM was performed on the mica surface, which can potentially affect the CYK4-dependent dynamic configuration of MKLP1 motor domains in an unexpected artificial manner. To examine whether CYK-4 binding can also reorganize the structure of ZEN-4 in solution, we performed Fӧrster resonance energy transfer (FRET) analysis by fluorescence anisotropy imaging microscopy (FAIM) (Fig 7A) [38,39]. To probe the conformational change in the neck region of the dimeric ZEN-4, the motor domains were replaced with yellow fluorescent protein (Venus) (VenusZ434-555, Fig 1A and 1E) [40]. The homo-FRET between the two Venus moieties is detected as the decrease of the fluorescence anisotropy (depolarization) (Fig 7A). To correct for fluorescence depolarization by the rotational diffusion of the whole molecule, which might also be affected by CYK-4 binding, the fluorescence anisotropy by excitation at 532 nm on the red edge of the absorption spectrum of Venus, which fails to cause homo-FRET, was also determined (Fig 7B and 7C). The FRET efficiency was calculated by comparing the fluorescence anisotropy by excitation at main band (Fig 7B) and at the red edge (Fig 7C) [41]. We observed a dramatic increase in the homo-FRET efficiency when the Venus-ZEN-4 neck dimer is in a heterotetrameric complex with C120 (Fig 7D). Consistent with our observations that the specific sequence of the CYK-4 coiled coil is dispensable both for the binding to ZEN-4 and for the effects on the ZEN-4 motor activity (Fig 5E), C40G also increased the homo-FRET efficiency similarly to C120 (Fig 7D). As homo-FRET reflects the intra-molecular geometry of the two Venus moieties, this indicates that C120 binding changes the relative positioning of the two Venus moieties in a dimer. This is consistent with a report by electron paramagnetic resonance [24] and confirms that the effect of CYK-4 on the configuration of the ZEN-4 motor domains observed by HS-AFM also occurs in solution. Because the ZEN-4 construct used for this analysis did not contain the motor domain, we conclude that CYK-4 changes the structure of the neck domain of the ZEN-4 dimer.

## Discussion

Here we demonstrated that CYK4 binding to the neck of MKLP1 modulates how the twin motor domains in heterotetrameric centralspindlin complex interact with microtubules. Specifically, we observed that CYK4 binding promotes antiparallel bundling of microtubules by MKLP1 and accumulation of the complex into the antiparallel microtubule overlap. Importantly, this is associated with a change in the dynamic inter-domain configuration between the two motor domains within the MKLP1 dimer. These data are consistent with, and further extend, preceding work on the molecular basis of central spindle formation and demonstrate well the power and versatility of HS-AFM that allows us to directly visualize structural dynamics of protein molecules in solution at sub-molecular spatial and sub-second temporal resolution.

Previously, we proposed a model for progressive accumulation of centralspindlin to the spindle midzone based on the spindle bipolarity and the positive feedback loop of centralspindlin-clustering and microtubule bundling under temporal and spatial regulation by mitotic kinases [34,36]. Although this model did not assign centralspindlin a preference for a specific type of bundling polarity, the preferential affinity of ZEN-4 for the antiparallel microtubule overlap, augmented by CYK-4 (Fig 4), would clearly be expected to contribute to the recruitment of centralspindlin to the spindle midzone, where antiparallel overlap of the interpolar microtubules exists even before the anaphase onset [42]. Furthermore, limiting accumulation on parallel microtubule bundles, such as kinetochore fibers, prevents mislocalization away from the central spindle. It will now be of great interest to discover how centralspindlin cooperates with PRC1, another microtubule-bundling factor crucial for the central spindle formation. PRC1 is homologous to yeast Ase1 and plant MAP65, which preferentially form antiparallel bundles [43–55].

In vivo experiments have shown that interaction between CYK-4 and ZEN-4 is critical for the formation of a robust central spindle [3,13]. This is consistent with our in vitro data showing that CYK-4 binding increases the affinity of ZEN-4 to microtubules and slows down ZEN-4 motility (Figs 4 and 5). Interestingly, we did not observe sliding of antiparallel microtubule bundles made by the CYK-4/ZEN-4 complex (Fig 4). This is in stark contrast with the sliding of antiparallel microtubule bundles by other mitotic kinesins in the kinesin-5 and -14 classes, which play important roles in formation of the metaphase bipolar spindle [54,56–60]. In the first cell division of *C*. *elegans* embryos, the whole spindle is under mechanical tension produced by a dynein-dependent “cortical pulling force” that pulls spindle poles towards the cortex by hauling on the astral microtubules [61–63]. This provides the major driving force for the anaphase B spindle elongation, with the central spindle working largely as a brake against this force [64]. The slower motility and stronger affinity of ZEN-4 when it is in a complex with CYK-4 might reflect an evolutionary adaptation of this class of kinesin superfamily molecules to its role as the mechanical bundler and, potentially, a brake, rather than a transporter or a microtubule sorter.

**Fig 8:**
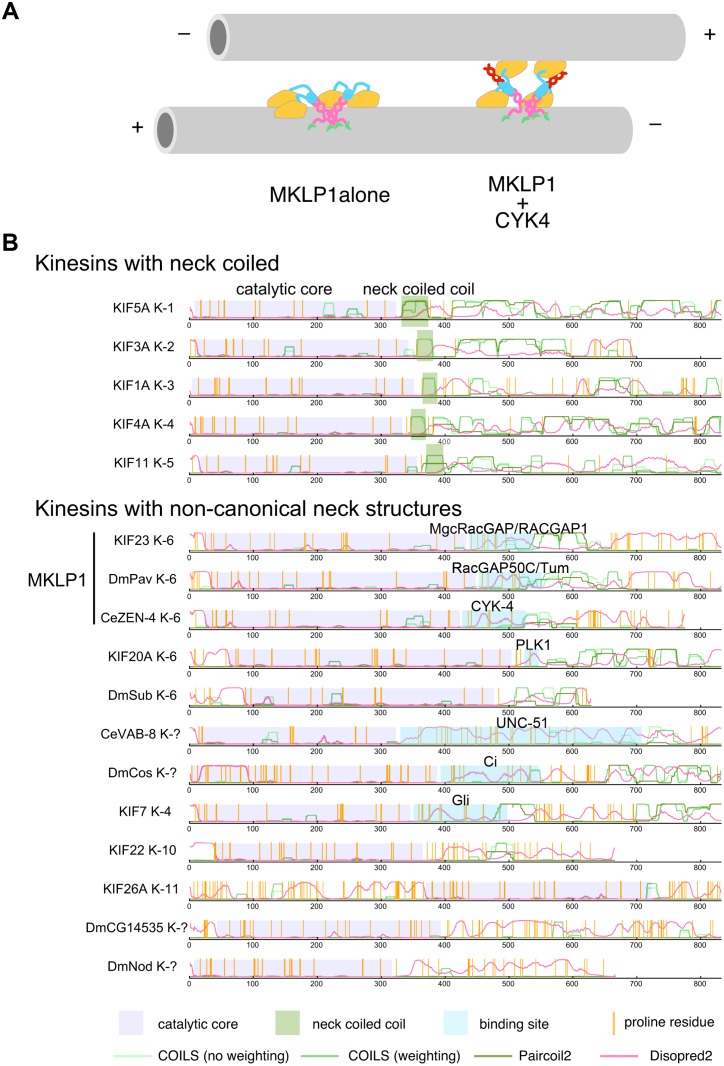
Model of microtubule bundling by ZEN-4-CYK-4 complex and its implications to other non-canonical N-kinesins. (A) An illustration depicting a model on how the CYK4 binding to the MKLP1/ZEN-4 neck modulates the interaction between MKLP1 and microtubules. In the absence of CYK4, both the MKLP1 motor domains in a dimer, which in turn forms a higher-order cluster (for simplicity, only two dimers are illustrated), can interact with a single microtubule using its long and flexible neck (left). CYK4 binding to the neck domain of MKLP1 restricts the relative configuration of the two motor heads into a 2-fold symmetry defined by the two parallel coiled coils. This makes it difficult for two heads in an MKLP1 dimer to simultaneously bind to the same microtubule, leaving one head in an optimal position to interact with a second microtubule in an antiparallel orientation (right). (B) A bioinformatic analysis identifies additional N-kinesins with a non-canonical neck sequence. Propensities of coiled coil formation by COILS [76] and Paircoil2 [77] and of disordered structures by DISOPRED [78] are plotted, along with the kinesin catalytic core defined by multiple sequence alignment and the positions of proline residues. Binding sites to known partners are also shown. In addition to the kinesin-6 family members, other N-kinesins also have a non-canonical, long non-coiled coil neck sequence, which in some cases has been shown to be a partner-binding site.

The flexible head-to-head coordination in the absence of CYK4 is consistent with the previous electron microscopic observation of dimeric ZEN-4 bound to microtubules [65], in which two motor domains in a single dimer bound to a microtubule in more variable positioning than observed in an Eg5 kinesin-5 dimer. Considering the 2-fold rotational symmetry that the parallel coiled coils of both CYK4 and MKLP1 should take, it is likely that CYK4 binding would fix the MKLP1 neck globular masses into the same symmetry around the linear molecular axis defined by their head-to-head association. This would result in a tendency that the two MKLP1 motor domains are positioned in the same 2-fold rotational symmetry, although there might still remain some level of flexibility due to the possible unstructured linker between the catalytic core and the neck globular domain. We speculate that the configuration of the two motor domains in 2-fold symmetry reinforced by CYK4 binding would prevent simultaneous binding of the two motor domains to the same single microtubule (Fig 8A). This would tend to facilitate antiparallel microtubule bundling, especially during the initial phase of bundling when the clustering is not yet extensive. The structural details of how the restriction in the configurations of the two motor domains in the MKLP1 dimer by CYK4 neck binding results in enhanced affinity and decreased motility remain unclear. Further experiments, including atomic-level structures of the CYK4-MKLP1 complex as well as structures of the motor complex dynamically interacting with its track [66,67], will be necessary to understand the mechanistic details of how the neck conformational change produced by CYK4 controls the MKLP1–microtubule interaction.

While most of the N-kinesins are likely to have a stereotypical neck structure that is similar to the short neck-linker followed by the neck coiled coil found in kinesin-1, some of them, in addition to the MKLP1 orthologs, do not follow this pattern and instead contain a long non-coiled coil sequence in their neck (Fig 8B and S1 Fig). Interestingly, the long neck sequences of these non-canonical N-kinesins seem to serve as a binding site for their binding partners. MKLP2/KIF20A, which is crucial to recruit chromosomal passenger complex to the spindle midzone during cytokinesis, is phosphorylated and bound by Polo-like kinase 1 in this region [68]. VAB-8 kinesin, which plays a role in cell migration and axonal pathfinding in *C*. *elegans* development, interacts with UNC-51 kinase through its long non-coiled coil neck region [69]. *Drosophila* Costal2 and mammalian KIF7 play crucial roles in the Hedgehog signaling pathway through interaction with transcription factors, Cubitus interruptus (Ci) and Gli proteins, respectively, which bind to the neck region of these kinesins [70]. Revealing whether and how the neck-binding proteins modulate the motor activity of these kinesins should provide new insights into the molecular mechanisms of these signaling pathways as well as the general mechanochemical principle of molecular motors.

## Materials and Methods

### Protein Expression and Purification

The ZEN-4 constructs were made using standard cloning techniques to insert ZEN-4 fragments into pCBD-TEV-BIO vector [37] to generate and fusion with an N-terminal chitin-binding domain (CBD) cleavable by Tobacco etch virus (TEV) protease. Bicistronic vectors were made by inserting the desired combination of CBD-TEV-ZEN-4 and CYK-4 fragments into a pGEX-6p based vector [71], resulting in CBD-ZEN-4 and GST-CYK-4 fusions, with protease sites (TEV and PreScission, respectively) between the affinity tag and target protein. The proteins were extracted in high salt buffer (250 mM NaCl, 10 mM HEPES pH 7.7, 1 mM EGTA, 1 mM MgCl_2_, 0.1% Triton X-100, 1 mM DTT, 0.1 mM ATP, 10 μg/ml leupeptin and 10 μg/ml pepstatin, 1 mM PMSF) and purified by affinity chromatography. CBD-tagged proteins were affinity purified with chitin beads, followed by elution with TEV protease. GST-tagged proteins were affinity purified with glutathione Sepharose beads and eluted with PreScission protease. In the case of ZEN-4/CYK-4 co-purification, the chitin beads were used first, and the eluted fractions were pooled and incubated with glutathione Sepharose beads. This ensured that the purified protein complex contained both ZEN-4 and CYK-4 subunits. Using a Superdex 200 10/300 GL gel filtration column, recombinant protein samples were finally purified and buffer exchanged into 250 mM NaCl, 10 mM HEPES pH 7.7, 1 mM EGTA, 1 mM MgCl_2_, 200 μM DTT, 25 μM ATP. For long-term storage, samples were frozen in liquid nitrogen and stored at -80°C. Human centralspindlin holocomplex was purified as described [34,36].

### High-Speed Atomic Force Microscopy

AFM imaging experiments were carried out using a laboratory-built high-speed atomic force microscope (HS-AFM) [28,30]. The sample was diluted to 1–3 nM with 25 mM KCl buffer (25 mM KCl, 1 mM MgCl_2_, 10 mM imidazole-HCl, pH 7.0, and 1 mM EGTA), which was used within 3 h. A droplet (2 μL) of the diluted sample was deposited on a freshly cleaved mica surface (~1 mm in diameter and <0.05 mm in thickness), which was beforehand glued on a glass stage (2 mm in diameter and 2 mm in height). After incubation for 3 min, to remove the molecules that were not attached to the mica surface, the sample surface was rinsed with 25 mM KCl buffer of ~20 μL without drying the sample surface. When we observed the molecule with 0 mM KCl buffer (1 mM MgCl_2_, 10 mM imidazole-HCl, pH 7.0, and 0.1 mM EGTA), the sample surface was additionally rinsed with 0 mM KCl buffer. Subsequently, the sample stage was immersed in a liquid cell filled with either 25 mM KCl buffer or 0 mM KCl buffer of ~60 μL in which a small cantilever was fixed. Imaging was carried out by HS-AFM in the tapping-mode. The detailed procedure related to the HS-AFM imaging experiments was recently summarized [72].

For image analysis, we used laboratory built software coded by Visual C# (Visual Studio 2010, Microsoft, Inc.). A low-pass filter and a flattening filter were applied to remove spike noise and to make the XY plane flat. The XY coordinate of the center of mass of a ZEN-4 motor domain was semi-automatically determined by the following steps. First, a point within the domain likely to be highest was manually specified. Second, the actual peak position was automatically determined within the area of 5×5 pixels around the manually specified point. Third, the center of mass was calculated as the weighted mean of the XY coordinates within the 5×5 pixel area around the actual peak, using their heights as weights.

### Microtubule Assays

Tubulin was purified from porcine brain by polymerization-depolymerization cycles followed by phosphocellulose chromatography [73] and modified with N-ethylmaleimide [74]. Biotin-, Cy5-, and rhodamine-labeled tubulins were purchased from Cytoskeleton Inc. Plus-end labeled microtubules were made as described.

For the microtubule sedimentation assays, glycerol-polymerized and taxol-stabilized microtubules were mixed with Z555, Z555ΔN, Z555/C120, or Z555ΔN/C120 and supplemented to 50 mM NaCl, 1 mM DTT, 10 μM taxol, and 2 mM AMP-PNP. Microtubules were sedimented by centrifugation in a 100 μl reaction mix using a Beckman TLA120.1 rotor (96,460 × g, 25°C, 20 min). Total, supernatant and pellet fractions were analyzed by SDS-PAGE.

Microscopy-based assays were performed at 20°C in flow cells (about 2.5 × 18 × 0.1 mm^3^ = 4.5 μl) made between a glass slide and a 18×18 mm^2^ coverslip using strips of double sided Scotch tape (3M) and observed by a CellR TIRFM total internal reflection fluorescence microscope system (Olympus) equipped with an iXon+ DU897 electron-multiplying (EM)-CCD camera (Andor). Images were processed with ImageJ (http://imagej.nih.gov/ij/) and data analyzed by R (http://www.r-project.org).

For microtubule gliding assays, ZEN-4-mAG was immobilized in anti-mAG antibody coated flow cells. Motility of rhodamine-labeled microtubules [75] was observed in motility solution (10 mM potassium PIPES pH 6.9, 25 mM KCl, 1 mM EGTA, 1 mM MgCl_2_, 1 mg/ml casein, 5 mM DTT, 1 mM ATP, 20 μM taxol, 4 mM DTT, 1 mg/ml glucose oxidase, 0.1 mg/ml catalase, and 10 mM glucose) by epifluorescence with Semrock Cy3-4040B filter set.

For ZEN-4 binding experiments, rhodamine-labeled microtubules were immobilized on coverslips by an anti-tubulin antibody (Sigma Aldrich, #T4026). ZEN-4-mAG complexes were perfused into the chamber in the above motility solution, with AMP-PNP substituted for ATP, and observed by total internal reflection fluorescence with a 488 nm laser and Chroma 41001 filter set. Quantification of ZEN-4 binding was achieved with ImageJ by measuring the fluorescence intensity along 2 μm of the microtubule, and subtracting the same measurement from an adjacent microtubule-free region (10–15 regions analyzed for each condition).

To observe microtubule bundling in solution, motor complexes were mixed with microtubules in motility solution and perfused into flow cells for observation. To observe ZEN-4 behavior on microtubule overlaps with defined polarities, biotinylated and dimly rhodamine-labeled microtubules (MT1) was first immobilized into a flow cell sequentially coated with biotin-BSA and Neutravidin (Pierce). Motility solution containing motor complexes and polarity labeled bright-rhodamine labeled microtubules (MT2) [49] was then perfused into the flow cell. Microtubule overlaps were observed by epifluorescence. Z601mAG movement and accumulation were observed by TIRF microscopy.

### Fӧrster Resonance Energy Transfer

Aliquots of VenusZ435-555 preparations complexed with or without CYK-4 fragments were put on a glass coverslip containing a press-to-seal (Life Technologies Ltd., Paisley, United Kingdom) silicone isolator and covered with another glass coverslip to avoid evaporation during measurements. Repeated widefield epifluorescence anisotropy images of each solution were obtained using a modified Nikon eclipse TE 300 inverted total internal reflection (TIRF) microscope (Nikon Ltd. UK, Kingston upon Thames, United Kingdom), with a 491 nm laser (Cobolt Calypso, 100 mW) for main band illumination and a 532 nm (30 mW) laser combined with an excitation filter (FL05532-1-1, Semrock, Rochester, NY, United States) for red edge illumination. Polarization-resolved fluorescence images were obtained with a 1.47 NA TIRF objective, a Semrock Di02-R532, a BP 545–580 nm emission filter, and a polarization beam splitter which separates the polarization components onto distinct areas of an EM-CCD camera (Andor iXon DV885, Belfast, United Kingdom). For each measurement, the EM-CCD was set to −50°C, 50 ms exposure time, constant EM gain, and to average 100 frames to minimize noise. The fluorescence anisotropy was evaluated by processing the raw image data in MATLAB (The MathWorks Inc., Natick, MA, United States): the polarization images were registered; dark current background was subtracted; the reference fluorescence measurement of the buffer solution was subtracted (affecting the final anisotropy values by less than 0.001), and the anisotropy of the samples was determined using Equation 1.

$$r=\frac{I_{∥}-GI_{⊥}}{I_{∥}+2GI_{⊥}}$$ (1)

G accounts for the ratio of the intensity response of the two detector channels, determined using a dilute aqueous dye solution (1 mM Oregon Green in ddH2O, Life Technologies) with a known fluorescence anisotropy of zero. The anisotropy *r* of each sample was evaluated on a pixelwise basis from the measured intensities (*I*) of fluorescence light with polarization parallel (*I*_||_) and perpendicular (*I*_⊥_) to the illumination polarization. The mean value of this anisotropy over the (uniform) image of the dye solution provides a single measurement of the sample fluorescence anisotropy; this measurement was repeated three times for each sample to determine instrument precision, and two such sets were taken for each solution to determine the uncertainty of fluorescence anisotropy due to solution variation. As a whole, the fluorescence anisotropy was determined with an instrument error better than 0.001 and with a total measurement uncertainty for repeated samples better than 0.004.

The average FRET efficiency of the Venus fluorescent protein in each solution was determined using the “red edge and main band” method [41], which can separate the effect of FRET from any effect of rotational diffusion on measured fluorescence anisotropy. The FRET efficiency was calculated by 1—*r/r0*, where *r* is the main band anisotropy and *r0* is the red edge anisotropy. ANOVA was performed by R with correction for multiple comparisons by Tukey’s method.

## Supporting Information

S1 Data The numerical data used for Figs 4D and 4E; 5D, 5E, and 5G; 6B–6E; 7; S4A–S4E; and S5B; and the X, Y, Z coordinates of AFM images in Figs 2, 3, 6, and S3. (XLSX)

S1 Fig Alignment of N-kinesins from human (Hs), fly (Dm), nematode (Ce), and budding (Sc) and fission (Sp) yeasts. The sequences of the catalytic core corresponding to the α5-β8-α6 and the neck linker sequences of the canonical N- kinesins were aligned by ClustalX. The neck coiled coil sequences of the canonical N-kinesins were aligned manually based on the heptad register assignment predicted by COILS and Paircoils2. Coloring follows the default scheme of ClustalX.
(EPS)

S2 Fig SDS-PAGE analysis of Superdex200 size-exclusion chromatography as the final step of purification of Z775mAG/C120 (A), Z555/C120 (B), Z601/C40G (C) and Venus-Z435-555/C120 (D). Note that the ZEN-4 and CYK-4 fragments were co-eluted from the column as a single peak.
(TIF)

S3 Fig A panel of HS-AFM images of independent molecules *C*. *elegans* centralspindlin constructs taken from a panel of 5 s movie clips (S7 Movie, S8 Movie, and S9 Movie, respectively). The pseudocolored images were from the molecules shown in Fig 2. Bar, 20 nm.
(EPS)

S4 Fig (A–C) Time courses of the head-to-head distance of ZEN-4 motor domains (D_H-H_) before and after C120 dissociation (other cases). Blue and red lines represent the time course of D_H-H_ before and after C120 dissociation, respectively. These time courses were obtained from the molecules observed in S9 Movie. (D) Summary of geometrical parameters of Z775mAG/C120. (E) Summary of head-to-head distance measurements.
(EPS)

S5 Fig Quantification of stoichiometry of ZEN-4 and CYK-4 in YFP-ZNCC/C120 complex by SDS-PAGE and densitometry. Intensities of the bands of YFP-ZNCC ZEN-4 (“Z”) and C120 CYK-4 fragments (“C”) in a SDS-PAGE gel (A) were measured and the ratio of the intensities (Z/C) were plotted. Assuming staining proportional to the molecular weight, the average intensity ratio 3.1 ± 0.1 corresponds to a molar ratio of Z:C = 1:0.94.
(EPS)

S1 Movie QuickTime movie of Z775mAG observed by HS-AFM at low salt. Detailed specifications are found in S1 Text.
(MOV)

S2 Movie QuickTime movie of Z775mAG observed by HS-AFM. Detailed specifications are found in S1 Text.
(MOV)

S3 Movie QuickTime movie of Z601 observed by HS-AFM. Detailed specifications are found in S1 Text.
(MOV)

S4 Movie QuickTime movie of Z775mAG/C120 observed by HS-AFM. Detailed specifications are found in S1 Text.
(MOV)

S5 Movie QuickTime movie of Z601/C120 observed by HS-AFM. Detailed specifications are found in S1 Text.
(MOV)

S6 Movie QuickTime movie of Z601/C40G observed by HS-AFM. Detailed specifications are found in S1 Text.
(MOV)

S7 Movie QuickTime movie of HS-AFM images of *C*. *elegans* centralspindlin constructs shown in S3 Fig. Detailed specifications are found in S1 Text.
(MOV)

S8 Movie QuickTime movie of human centralspindlin holocomplex observed by HS-AFM. Detailed specifications are found in S1 Text.
(MOV)

S9 Movie QuickTime movie of human centralspindlin holocomplex observed by HS-AFM. Detailed specifications are found in S1 Text.
(MOV)

S10 Movie QuickTime movie of dissociation of Z775mAG/C120 observed by HS-AFM. Detailed specifications are found in S1 Text.
(MOV)

S11 Movie QuickTime movie of dissociation of Z775mAG/C120 observed by HS-AFM. Detailed specifications are found in S1 Text.
(MOV)

S1 Text Detailed specifications for S1–S11 Movies. (DOCX)

## Abbreviations

AFM: Atomic force microscopy
CC: coiled coil
Ci: Cubitus interruptus
D_H-H_: head-to-head distance
FAIM: fluorescence anisotropy imaging microscopy
FRET: Förster resonance energy transfer
HS-AFM: high-speed AFM
*K*_d_: dissociation constant
MT: microtubule
