# Supplementary figures and images for: CYK4 Promotes Antiparallel Microtubule Bundling by Optimizing MKLP1 Neck Conformation
Source: PLoS Biol. 2015 Apr 13;13(4):e1002121. doi: 10.1371/journal.pbio.1002121 (PMC4395295; doi:10.1371/journal.pbio.1002121)

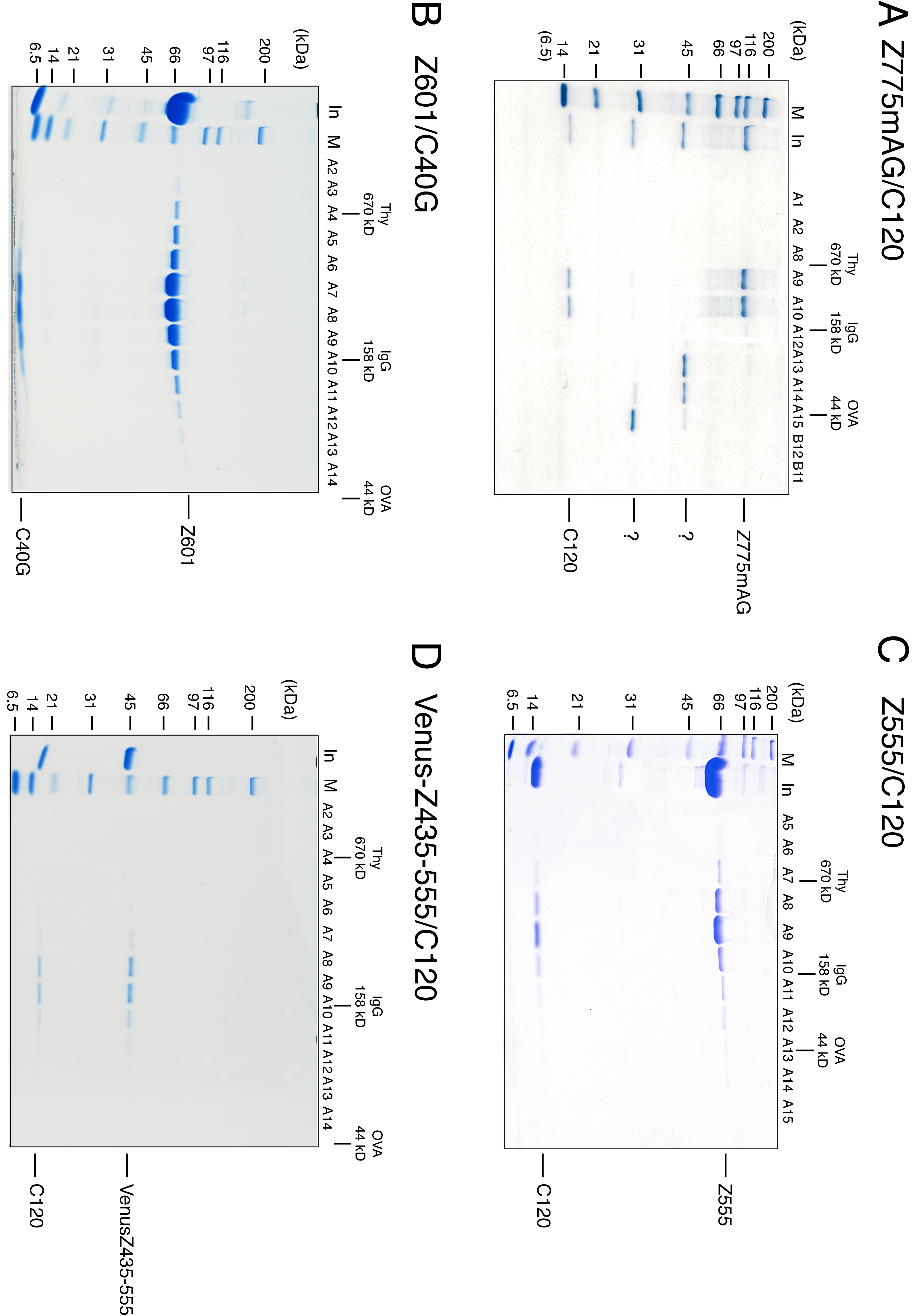

Supplement: S2 Fig — Note that the ZEN-4 and CYK-4 fragments were co-eluted from the column as a single peak. (TIF) [file pbio.1002121.s003.tif]
