# Supplementary material for: CYK4 Promotes Antiparallel Microtubule Bundling by Optimizing MKLP1 Neck Conformation
Source: PLoS Biol. 2015 Apr 13;13(4):e1002121. doi: 10.1371/journal.pbio.1002121 (PMC4395295; doi:10.1371/journal.pbio.1002121)
Supplement: S1 Text — (DOCX) [file pbio.1002121.s018.docx]

**Supplementary Movies**

QuickTime movies of HS-AFM images. Detailed specifications are as below.

| **Title** | **Assoc-iated to** | **Sample** | **File** | **Size**  **(pixels)** | **Size**  **(nm)** | **Cycle time (ms/ frame)** | **Replay**  **speed** | **z-scale**  **(nm)** |
| --- | --- | --- | --- | --- | --- | --- | --- | --- |
| S1 Movie | Fig. 2A | Z775mAG (no salt) | S1_movie.mov | 80 | 70 | 149 | 1x | 4.0 |
| S2 Movie | Fig. 2B | Z775mAG | S2_movie.mov | 80 | 70 | 100 | 1x | 4.5 |
| S3 Movie | Fig. 2C | Z601 | S3_movie.mov | 80 | 60 | 100 | 1x | 4.5 |
| S4 Movie | Fig. 2D | Z775mAG/C120 | S4_movie.mov | 80 | 60 | 200 | 1x | 4.5 |
| S5 Movie | Fig. 2E | Z601/C120 | S5_movie.mov | 80 | 60 | 100 | 1x | 4.5 |
| S6 Movie | Fig. 2F | Z601/C40G | S6_movie.mov | 80 | 60 | 100 | 1x | 4.5 |
| S7 Movie | S3 Fig. | *C.elegans* proteins* | S7_movie.mov | variable | 60 | as above | 1x | as above |
| S8 Movie | Fig. 3 | human holocomplex | S8_movie.mov | 80 | 80 | 215 | 1x | 4.5 |
| S9 Movie | Fig. 3 | human holocomplex* | S9_movie.mov | 80 | 80 | 215 | 1x | 4.5 |
| S10 Movie | Fig. 5A | Z775mAG/C120 dissociation ** | S10_movie.mov | 80 | 60 | 140 | 1x | 4.5 |
| S11 Movie | S4 Fig. | Z775mAG/C120 dissociation (A, B) * | S11_movie.mov | 80 | 80 | 104 | 1.04x | 4.5 |
|  |  | Z775mAG/C120 dissociation (C) * |  | 64 | 60 | 100 | 1x | 4.5 |

(*) The pseudo colored images are identical to those in corresponding movies (S1 to S6 and S8 Movies, respectively.

(**) The colored triangle at the lower left corner indicates that the frame is before (blue) or after (red) dissociation of C120.
